# The degradation of Rap1GAP via E6AP-mediated ubiquitin-proteasome pathway is associated with HPV16/18-infection in cervical cancer cells

**DOI:** 10.1186/s13027-021-00409-9

**Published:** 2021-12-24

**Authors:** Yinghui Wang, Yihang Xie, Boxuan Sun, Yuwei Guo, Ling Song, Dawit Eman Mohammednur, Chunyan Zhao

**Affiliations:** 1 College of Laboratory Medicine, Dalian Medical University, 9 West Section, Lvshun South Road, Dalian, Liaoning, China; 2 Present Address: Liaoning Provincial Center for Disease Control and Prevention, Shenyang, China; 3 Present Address: Foruth Teaching Hospital, Tianjin University of Traditional Chinese Medicine, Tianjin, China

**Keywords:** Cervical cancer, Ubiquitin-proteasome pathway, Autophagy, Rap1GAP, HPV, E6AP

## Abstract

**Background:**

Cervical cancers are closely associated with persistent high-risk human papillomaviruses (HR HPV) infection. The main mechanism involves the targeting of tumor suppressors, such as p53 and pRB, for degradation by HR HPV-encoded oncoproteins, thereby leading to tumorigenesis. Rap1GAP, a tumor suppressor gene, is down-regulated in many cancers. Previous studies have revealed that down-regulation of Rap1GAP is correlated with HPV16/18 infection in cervical cancer. However, the molecular mechanism remains unclear. In this study, we aimed to address the degradation pathway of Rap1GAP in HPV-positive cervical cancer cells.

**Methods:**

HPV-positive (HeLa and SiHa) and negative (C33A) cervical cancer cells were used to analyze the pathways of Rap1GAP degradation. MG132 (carbobenzoxy-leucyl-leucyl-leucine) was used to inhibit protein degradation by proteasome. Co-immunoprecipitation (co-IP) was used to detect the interaction between Rap1GAP and E6AP. siRNA for E6AP was used to silence the expression of E6AP. Rapamycin was used to induce cell autophagy. Western blotting was used to check the levels of proteins.

**Results:**

Following treatment with MG132, the levels of Rap1GAP were increased in the HR HPV-positive HeLa and SiHa cells, but not in the HPV-negative C33A cells. Co-immunoprecipitation assay revealed ubiquitinated Rap1GAP protein in HeLa and SiHa cells, but not in C33A cells. E6-associated protein (E6AP) mediated the ubiquitination of Rap1GAP by binding to it in HeLa and SiHa cells, but not in C33A cells. However, the levels of Rap1GAP were decreased in HeLa and SiHa cells after knocking down E6AP by siRNA. Silencing of E6AP did not affect the levels of Rap1GAP in C33A cells. Autophagy marker p62 was decreased and LC3 II/LC3 I was increased after knocking down E6AP in HeLa cells, but not in C33A cells. The levels of Rap1GAP were decreased after treating the cells with rapamycin to induce cell autophagy in HeLa and C33A cells.

**Conclusion:**

Rap1GAP may be degraded by autophagy in cervical cancer cells, but HPV infection can switch the degradation pathway from autophagy to E6AP-mediated ubiquitin-proteasome degradation. E6AP may be a key component of the switch.

## Background

Cervical cancer is the second most prevalent malignant tumor in women based on its incidence and mortality rate [1], and has become a serious public health problem worldwide. Persistent infection with high-risk human papillomaviruses (HR HPVs) is responsible for 99% of cervical cancers [2, 3]. HPVs, the small double-stranded DNA viruses, are clinically grouped into “high-” and “low-risk” based on their propensity for malignant progression [4]. More than 200 different types of HPVs have been characterized thus far [5], of which, HPV16 and HPV18 are the two major high-risk oncogenic viruses that are most closely associated with cervical cancer. They account for approximately 70% of all cervical cancers [2, 5]. In one of the major mechanisms of HR HPV-mediated cancer, the oncoproteins encoded by HR HPV, especially E6 and E7, target tumor suppressors such as p53 and pRB, respectively, for degradation through the ubiquitin-proteasome system (UPS) [6]. Recently, several such cellular protein targets of the viral oncoproteins have been identified that show significant effects on tumorigenesis [7].

Ras-related regulatory protein 1 GTPase-activating protein (Rap1GAP) is a GTPase-activating protein of the small G protein Rap1. As a crucial tumor suppressor, it inhibits cell proliferation, migration, and invasion [8–12]. It is found that Rap1GAP was down-regulated in many cancers, such as breast cancer [13], squamous cell carcinoma [10, 12, 14, 15], pancreatic cancer [11], renal cell carcinoma [8], thyroid cancer [7, 16–18], gastric cancer [19, 20], and colorectal cancer [21]. The lower level of Rap1GAP is thought to be associated with the size of the tumor, the advanced tumor-node-metastasis, and indicates a worse prognosis [20]. Currently, the known mechanisms of Rap1GAP down-regulation include loss of gene heterozygosity [22], promoter methylation [7], and gene mutation [23]. In our previous studies on cervical cancer, we found that Rap1GAP inhibits the migration and invasion of cervical cancer cells. The expression of Rap1GAP was found to be decreased in cervical cancer tissues and was negatively correlated with HPV16/18 infection [24]. In this study, we sought to better understand the mechanism of Rap1GAP down-regulation in cervical carcinogenesis and the role of HR HPV16/18 in the regulation of Rap1GAP levels.

## Methods

### Cell lines and antibodies

The HeLa cell line was purchased from Shanghai Institute of Biochemistry and Cell Biology (Shanghai, China). The C33A cell line was purchased from Procell Life Science and Technology Co. Ltd. (Wuhan, China). The SiHa cell line was purchased from Shanghai Zhong Qiao Xin Zhou Biotechnology Co. Ltd. (Shanghai, China). The p53 rabbit polyclonal antibody (10442-1-AP) and the ubiquitin-protein ligase E3A (UBE3A) rabbit polyclonal antibody (10344-1-AP) were purchased from Proteintech Group (Chicago, IL, USA). The glyceraldehyde-3-phosphate dehydrogenase (GAPDH) rabbit polyclonal antibody (AP0063) was purchased from Bioworld Technology Inc. (Nanjing, China), the Rap1GAP (Y135) rabbit monoclonal antibody (ab32119) was from Abcam (Shanghai, China), the ubiquitin mouse monoclonal antibody (sc-8017) and the mouse anti-LC3 β monoclonal antibody (sc-376404) were from Santa Cruz Biotechnology Inc. (Dallas, TX, USA). The goat anti-mouse IgG (ZB-2305) and goat anti-rabbit IgG (ZB-2301) antibodies were purchased from ZSGB-BIO Technology Co. Ltd. (Beijing, China).

### Cell culture

HeLa cells were cultured in Dulbecco’s modified Eagle’s medium (DMEM) (GIBCO; Thermo Fisher Scientific Inc., Waltham, MA, USA), and SiHa and C33A cells were cultured in minimum essential medium (MEM) (Hyclone; GE Healthcare Bio-Sciences AB, Uppsala, Sweden) supplemented with 10% (v/v) fetal bovine serum (Sijiqing; Zhejiang Tianhang Biotechnology Co. Ltd. Hangzhou, China), 100 U/mL penicillin, and 100 μg/mL streptomycin (Hyclone; GE Healthcare Bio-Sciences AB.) routinely (37 °C, 5% CO_2_).

### Western blotting

Cells were lysed with RIPA lysis buffer (Beyotime Biotechnology, Shanghai, China) and centrifuged at 12,000*g* for 10 min at 4 °C. The supernatant was taken into a new tube as the total cell lysate. Protein concentration in the total cell lysate was assessed using the Bicinchoninic Acid Assay (P0010S) (Beyotime Biotechnology). A total of 20 μg of proteins was separated using 10% (15% for LC3) sodium dodecyl sulfate polyacrylamide gel electrophoresis (SDS-PAGE), and then the proteins were transferred to a polyvinylidene difluoride (PVDF) membrane (Merck Millipore, Merck KGaA, Darmstadt, Germany). The PVDF membrane was blocked with freshly prepared tris-buffered saline containing 5% skimmed milk powder for 2 h at about 25 °C, and then incubated with the primary antibody diluted in tris-buffered saline containing 5% milk overnight at 4 °C followed by incubation with the secondary antibody for 1 h at about 25 °C. The membrane was then incubated with BeyoECL Plus reagent (P0018S) (Beyotime Biotechnology) to detect the protein bands.

### Co-immunoprecipitation assay

Total cell lysates were incubated with the specific antibody at 4 °C overnight and then the supernatants were incubated with protein A + G agarose beads (Beyotime Biotechnology) at room temperature for 3 h. After three washes with PBS, the beads were re-suspended in 1 × SDS-PAGE loading buffer (Beyotime Biotechnology) and boiled for 5 min. The supernatants were then used for immunoblotting.

### MG132 treatment

Cells were plated in a 24-well plate at a density of 1.5 × 10^5^/well and cultured in DMEM for HeLa cells or MEM for SiHa and C33A cells routinely. Twenty-four hours later, the cells were treated with MG132 by replacing the complete medium with serum-free DMEM or MEM containing MG132 (dissolved with dimethyl sulfoxide) at a final concentration of 20 μmol/L for 24 h under the routine culture condition (37 °C, 5% CO_2_). The control cells were treated with the solvent of MG132 by replacing the complete medium with serum-free DMEM or MEM containing only dimethyl sulfoxide for 24 h under the same conditions.

### siRNA for E6AP

E6AP-specific siRNA sequence was 5′-CAACUCCUGCUCUGAGAUAtt and silencing control siRNA was 5′-UUCUCCGAACGUGUCACGUtt according to the papers [25, 26], and were synthesized by Suzhou Jima Gene Co., LTD (Suzhou, China). Cells were seeded in a 24-well plate at a density of 1.5 × 10^5^/well and cultured routinely. Eighteen hours later, the cells were transfected with siRNA with Lipofectamine TM 2000 (Thermo Fisher, Carlsbad, CA, USA) according to the manufacturer’s instruction. Forty-eight hours later, the cells were lysed and the lysates were collected to detect the protein levels by western blotting.

### Rapamycin treatment

Cells were seeded in 12-well plates at a density of 3 × 10^5^/well. Twenty-four hours later, the cells were treated with rapamycin at a final concentration of 20 nmol/L by culturing the cells in a medium with rapamycin. Seventy-two hours later, the cells were lysed and the lysates were collected for western blotting.

### Statistical analysis

Data are analyzed as mean ± standard deviation (SD) using the independent-samples t-test for comparing the means by SPSS 23.0. (SPSS Inc., Chicago, IL, USA). P < 0.05 was considered significant difference. GraphPad Prism 7 XML Project (GraphPad Software Inc., La Jolla, CA, USA) was used to create the illustrations.

## Results

### MG132 inhibits the degradation of Rap1GAP in HPV16/18 positive cervical cancer cells

**Fig. 1:**
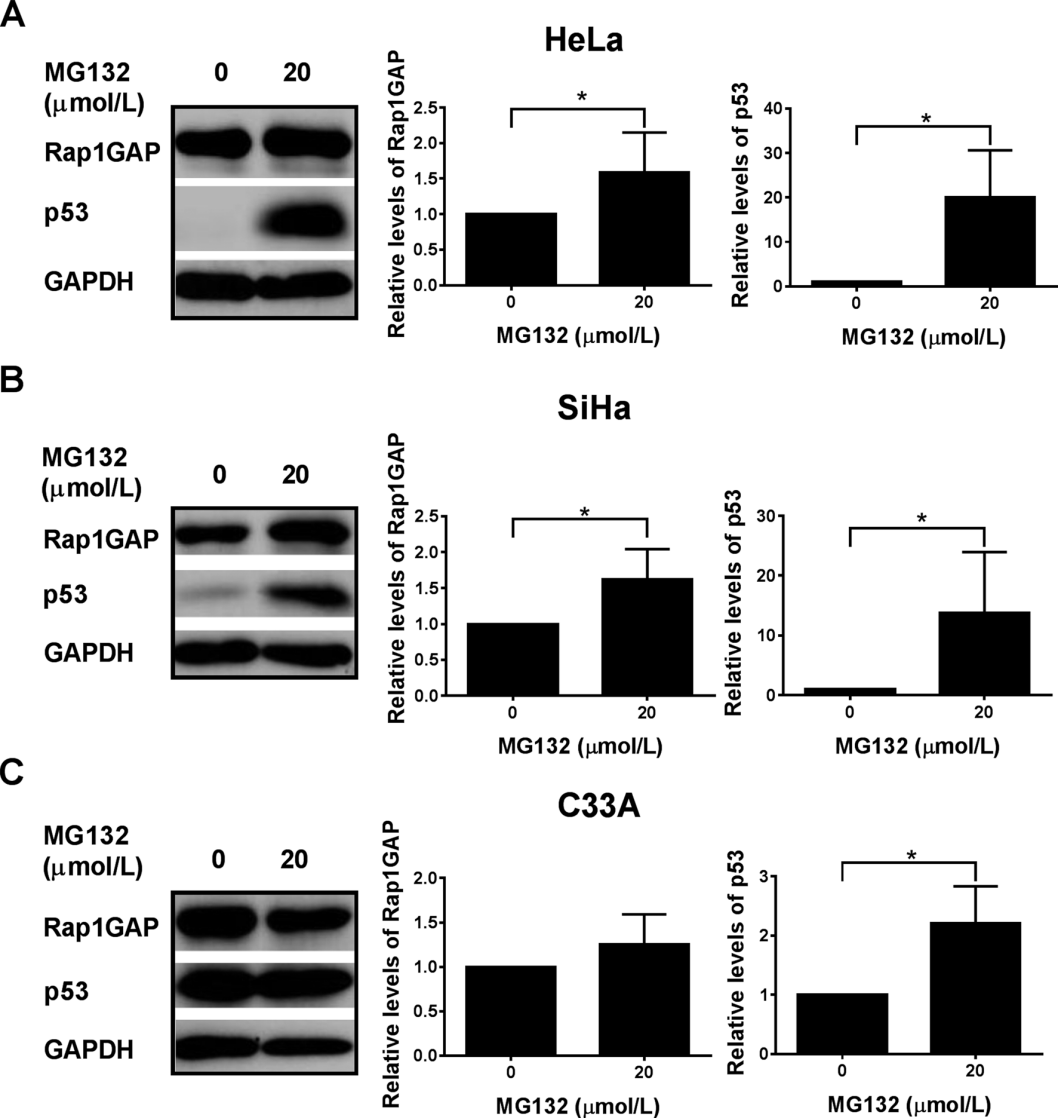
Degradation of Rap1GAP is inhibited by MG132 in HPV16/18 positive cervical cancer cells. HeLa (**A**), SiHa (**B**), and C33A (**C**) cells were treated with 20 μmol/L MG132 for 24 h. The levels of Rap1GAP and p53 proteins were analyzed using western blotting, as described in “Methods” section. The fold change is the ratio of the value from the cells treated with MG132 divided by that from the control cells. The data are from three independent experiments. *p < 0.05

The ubiquitin-proteasome pathway (UPP) is the major pathway for the degradation of cellular proteins [3]. HR HPV target proteins are mainly degraded by UPP [27]. To determine whether the down-regulation of Rap1GAP in cervical cancer cells was due to proteasome-mediated degradation, the cells were treated with a proteasome inhibitor, MG132, and the levels of Rap1GAP in the cells were analyzed before and after the treatment. To understand the role of HPV16/18 in the degradation of Rap1GAP, HPV16/18 positive cervical cancer cell lines, HeLa (containing HPV18) and SiHa (containing HPV1 6), and an HPV-negative cervical cancer cell line, C33A, were used in the study. The concentration of MG132 (20 μmol/L) used in this study was based on our previous study [28]. Following the treatment of cells with MG132 for 24 h, the relative levels of Rap1GAP were significantly increased in HeLa and SiHa (P < 0.05) (Fig. 1A, B), but not in C33A (P > 0.05) (Fig. 1C). To ensure the reliability of the results, p53, which is known to be degraded by UPP [29–33], was used as a positive control. The relative levels of p53 were significantly increased following MG132 treatment in all the three cell lines (p < 0.05) (Fig. 1), in agreement with other studies [30, 32, 34–37]. These results indicate that, unlike p53, the degradation of Rap1GAP inhibited by MG132 is associated with HPV16/18 infection.

### Rap1GAP is degraded by UPP in HPV16/18 positive cervical cancer cells, but not in HPV-negative cells

**Fig. 2:**
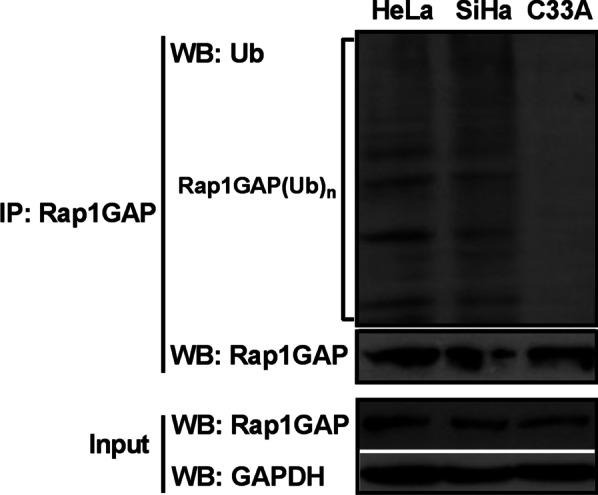
Rap1GAP is degraded by the UPP in HPV16/18 positive cervical cancer cells. Total cell lysates (as indicated) were used to detect the expression of Rap1GAP in the cells (input) and subjected to immunoprecipitation with an anti-Rap1GAP antibody. The pulled-down proteins were analyzed using immunoblotting with the anti-ubiquitin antibody to detect the interaction between Rap1GAP and ubiquitin. To avoid the disturbance bands at 55 kD and 25 kD formed by the antibody used for pull-down in co-IP, the anti-Rap1GAP antibody was from rabbit and the anti-ubiquitin antibody was from mouse in co-IP (see “Methods” section)

To provide direct evidence for Rap1GAP degradation through UPP, the interaction between Rap1GAP and ubiquitin was analyzed in HPV16/18 positive and negative cells using co-IP assays. Cell lysates were prepared from HeLa, SiHa, and C33A cells and an anti-Rap1GAP antibody was used for immunoblotting. Poly-ubiquitinated Rap1GAP (Rap1GAP[Ub]n) was found in HeLa and SiHa cells, but not in C33A cells (Fig. 2). These results confirmed that Rap1GAP was degraded by UPP in an HPV16/18-dependent manner.

### E6AP is involved in the ubiquitin-mediated degradation of Rap1GAP in HPV16/18 positive cervical cancer cells

**Fig. 3:**
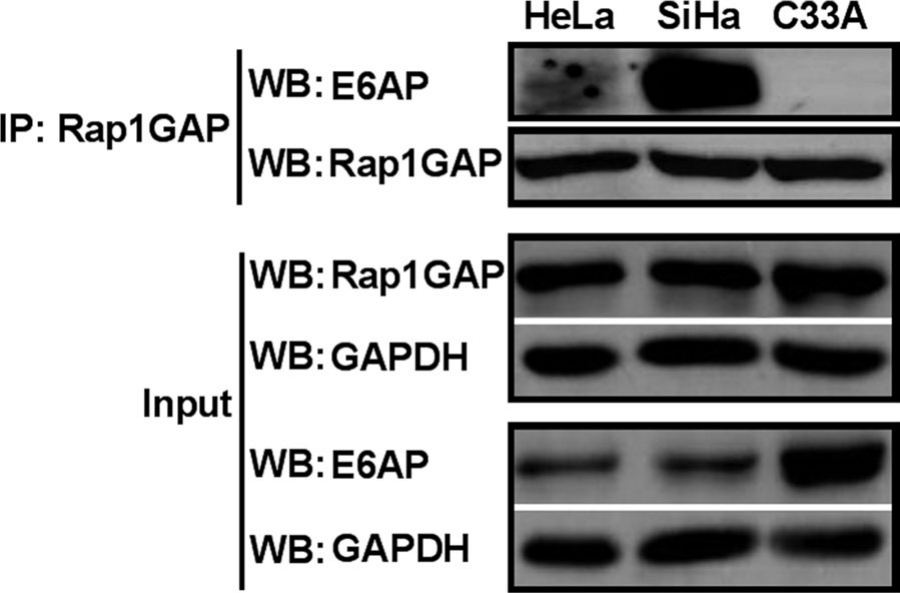
Rap1GAP interacts with E6AP in HPV16/18 positive cervical cancer cells. Total cell lysates from HeLa, SiHa and C33A cells were used to detect the expression of Rap1GAP and E6AP in the cells (input). Then, the lysates were subjected to immunoprecipitation with an anti-Rap1GAP antibody. The pulled-down proteins were analyzed using immunoblotting. Interaction between Rap1GAP and E6AP was detected with an anti-E6AP antibody, and the immunoprecipitation efficiency of Rap1GAP was verified with an anti-Rap1GAP antibody

E6-associated protein (E6AP) is an important E3 ubiquitin ligase. It plays a crucial role in HR HPV-associated cancers [6]. Because the ubiquitin-mediated degradation of Rap1GAP was found to be HR HPV16/18-dependent, we analyzed the interaction between Rap1GAP and E6AP using co-IP assays in HPV-positive cervical cancer cells. The results showed that Rap1GAP directly binds to E6AP in HeLa and SiHa cells, but not in C33A cells (Fig. 3), suggesting that E6AP is involved in the ubiquitin-mediated degradation of Rap1GAP in an HPV-dependent manner.

### Knocking down E6AP actives autophagy degradation pathway of Rap1GAP in HPV16/18 positive cervical cancer cells

**Fig. 4:**
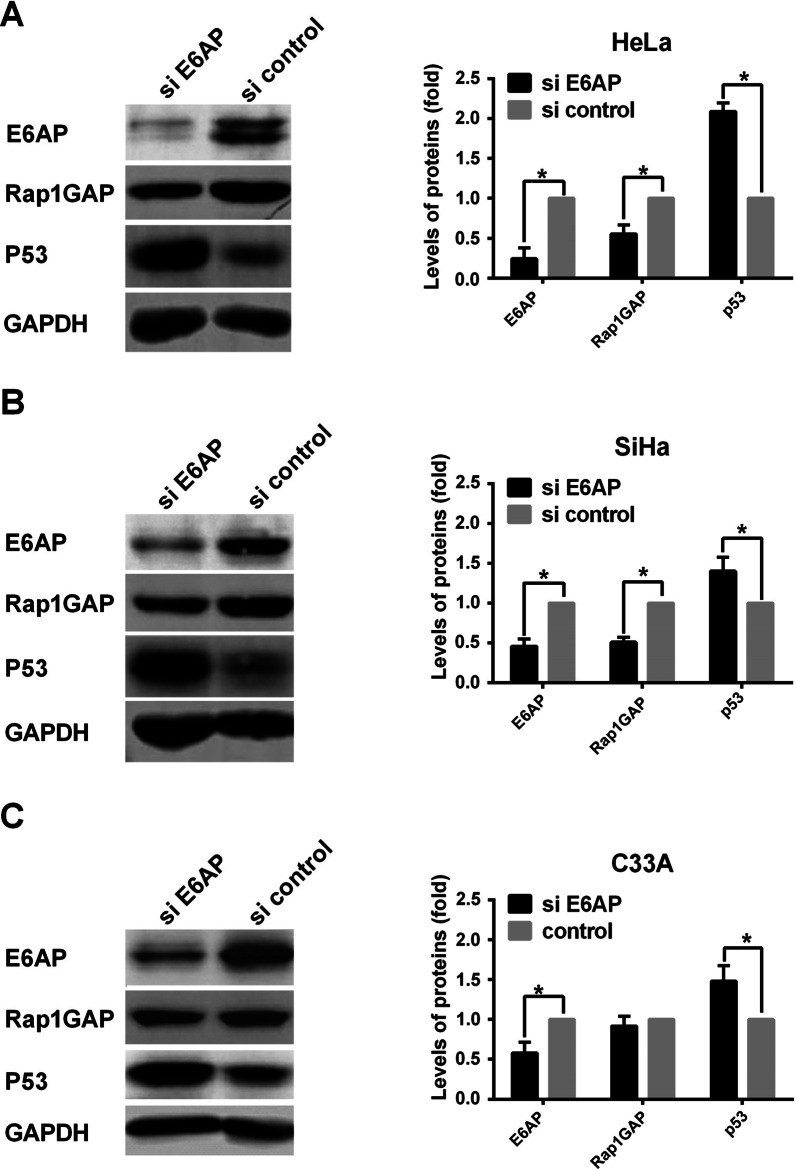
Knocking down E6AP increase the levels of Rap1GAP in HPV-positive cervical cancer cells. HeLa, SiHa and C33A cells were transfected with siRNA for E6AP, and the levels of E6AP were detected by western blotting to check the efficiency of knocking down. Then, the levels of Rap1GAP and p53 were detected by western blotting after silencing E6AP. *p < 0.05

To understand the role of E6AP in the ubiquitin-mediated degradation of Rap1GAP, we knocked down the expression of E6AP by siRNA in HeLa, SiHa and C33A cells. Surprisingly, the levels of Rap1GAP were decreased in HeLa and SiHa cells, but not in C33A cells. In C33A cells, the levels of Rap1GAP were not affected by silencing E6AP (Fig. 4). To confirm the reliability of the results, the levels of p53 were measured in the cells simultaneously. The results showed that the levels of p53 were elevated in the cells after E6AP was knocked down (Fig. 4).

**Fig. 5:**
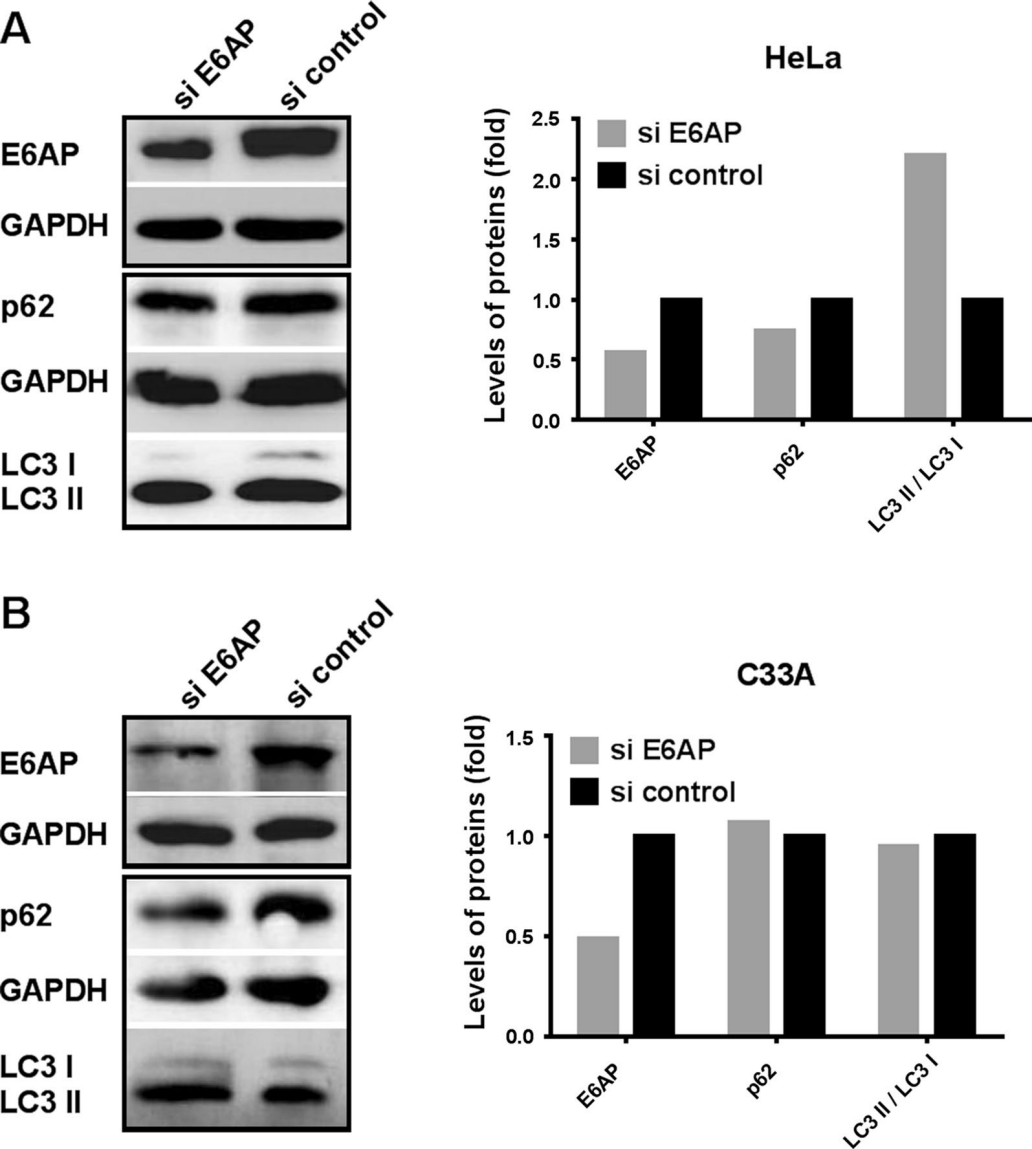
Knocking down E6AP activates the autophagy degradation pathway of Rap1GAP in HPV-positive cervical cancer cells. Cells, as indicated, were transfected with siRNA for E6AP to silence the expression of E6AP. The knocking-down efficiency was evaluated by detecting the levels of E6AP in the cells. Then, the autophagy markers p62 and LC3 II/LC3 I were checked to assess the levels of autophagy

**Fig. 6:**
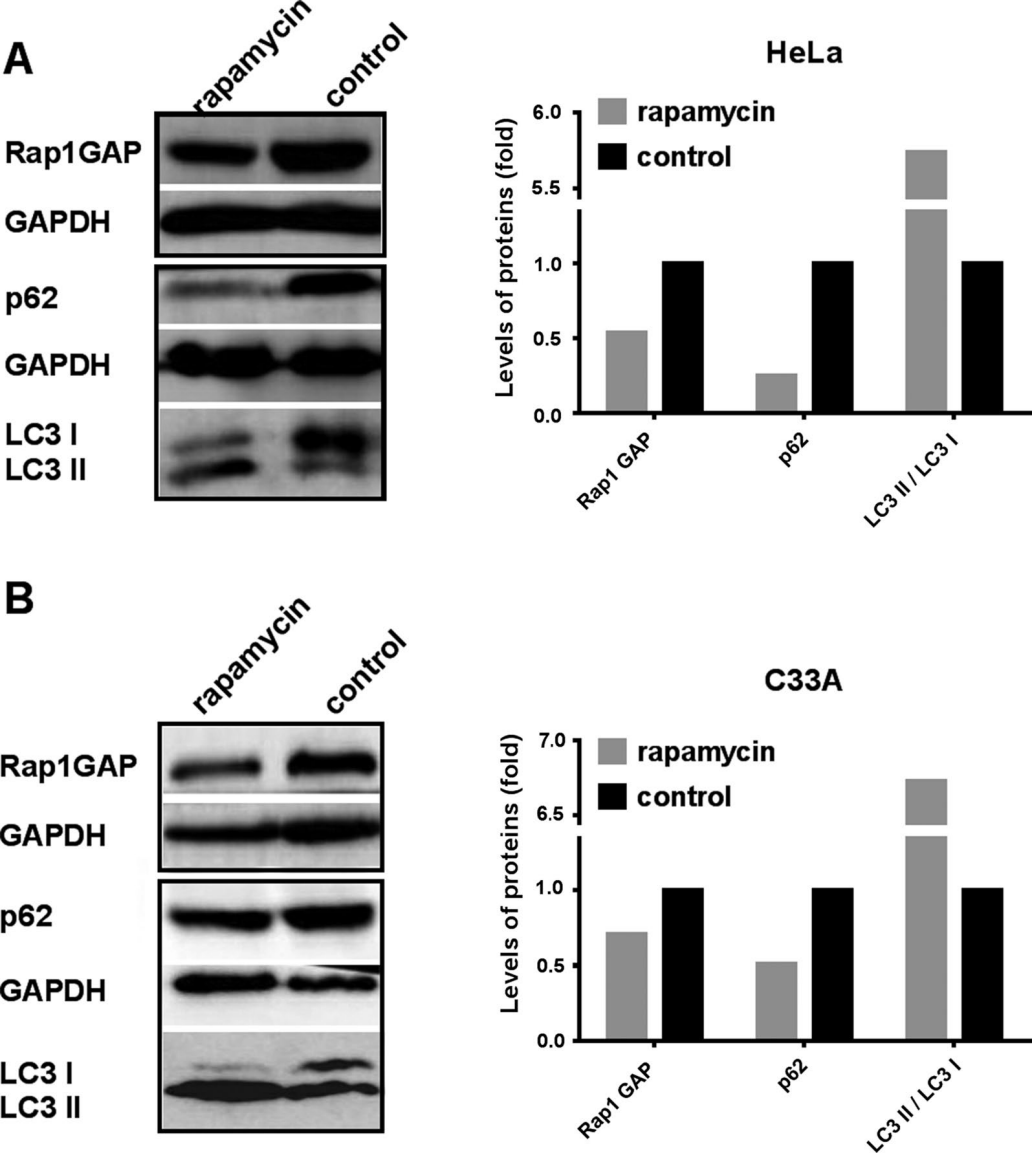
Rap1GAP is degraded by the autophagy pathway. Cells, as indicated, were treated with rapamycin for 72 h to induce cell autophagy. The cell lysates were used to check the autophagy markers p62 and LC3 II/I to assess the levels of autophagy. Then, the levels of Rap1GAP were detected by western blotting to check the effect of autophagy on the degradation of Rap1GAP

The autophagy-lysosome pathway (ALP) is another important pathway for protein degradation. There are complex cross-talks between UPP and ALP. To understand the phenomenon that down-regulation of E6AP decreased the levels of Rap1GAP, we explored whether knocking down E6AP activates the autophagy pathway, which leads to the degradation of Rap1GAP. E6AP was knocked down with siRNA for E6AP, the autophagy markers, p62 and LC3 II/LC3 I, were detected. The results showed that the levels of p62 were decreased and LC3 II/LC3 I was increased after E6AP was knocked down in HeLa cells. The levels of the autophagy markers were not affected in C33A cells (Fig. 5). The results indicate that down-regulation of E6AP can induce autophagy in HPV-positive cells but not in HPV-negative cells. To confirm whether the levels of Rap1GAP can be degraded by autophagy, we treated the cells with rapamycin to induce autophagy. The levels of Rap1GAP were decreased in HeLa and C33A cells as expected (Fig. 6). The results suggest that the decrease of Rap1GAP caused by knocking down E6AP may be due to the activation of ALP.

## Discussion

Ubiquitin-mediated degradation of proteins associated with HR HPVs plays a key role in HPV-related carcinogenesis. In addition to p53 and pRB, two important tumor suppressors, several target proteins of HR HPV oncoproteins have been discovered. Identification of HR HPV target proteins is of considerable significance not only to understand the molecular mechanism of tumorigenesis but also because they can be potential new targets for cancer therapy. Rap1GAP, a key molecule that regulates the activity of Rap1, was found to be down-regulated in cervical cancer with HPV16/18 infection. To investigate the mechanism of Rap1GAP down-regulation related to HPV16/18 infection in cervical cancer, the HR HPV positive cell lines, HeLa (containing HPV18) and SiHa (containing HPV16), and the HPV-negative cervical cancer cell line, C33A, were used in the study. Treatment of the cells with MG132 led to an increase in the protein levels of Rap1GAP in the HPV-positive cervical cancer cells, HeLa and SiHa, but not in the HPV-negative cervical cancer cell line, C33A (Fig. 1). Poly-ubiquitinated Rap1GAP was detected in HeLa and SiHa cells, but not in C33A cells (Fig. 2). These results suggested that ubiquitin-mediated degradation of Rap1GAP was associated with HR HPV infection. HPV16/18 infection likely triggers the degradation of Rap1GAP via UPP, which leads to the down-regulation of Rap1GAP in HPV16/18 infected cells.

Protein ubiquitination is catalyzed by a cascade of enzymes including the E1 Ub-activating enzymes, E2 Ub-conjugating enzymes, and E3 ubiquitin (Ub) ligases. E3 Ub ligases are responsible for substrate specificity by specifically recognizing the ubiquitinated target proteins. E3s are classified into three dominant groups, the homologous to E6AP C terminus (HECT), the Really Interesting New Gene (RING), and the RING-between-RING (RBR) [38]. E6AP belongs to the HECT type E3 with a signature HECT domain for E2 binding [39]. It is a 98 kDa protein encoded by the *UBE3A* gene, and therefore, it is also known as UBE3A. E6AP/UBE3A is widely expressed in cells. It is known for its important role in HR HPV-mediated p53 ubiquitin-proteasome degradation [27, 39, 40]. E6AP-mediated proteolysis plays an important role in many cellular processes associated with HPV-induced carcinogenesis [6, 39]. Therefore, the involvement of E6AP in the degradation of Rap1GAP by UPP was investigated. Due to E6AP itself is degraded by UPP, which can be promoted by HR HPV, the levels of E6AP are low in HPV-positive HeLa and SiHa cells (Fig. 3), in agreement with other studies [41]. An interaction between Rap1GAP and E6AP was found in HeLa and SiHa cells by co-IP assays (Fig. 3), indicating that E6AP is involved in HPV16/18-associated ubiquitination and degradation of Rap1GAP.

If E6AP is an E3 ligase of Rap1GAP degradation, the levels of Rap1GAP will increase after down-regulation of E6AP. Then we silenced E6AP by siRNA. Surprisingly, the levels of Rap1GAP were decreased in HeLa and SiHa cells (Fig. 4). To ensure the authenticity of the results, p53 was used as a control due to the reason that p53 is a well-known protein degraded by E6AP-mediated UPP in HPV-positive cervical cancer cells [42, 43]. The levels of p53 were increased after E6AP was knocked down in HeLa and SiHa cells (Fig. 4), consistent with the results of other researches [44]. In HPV-negative C33A cells, the levels of p53 were also increased after knocking down E6AP, which is different from that in other HPV-negative cells such as MCF7 and 293 T (data not shown). This may be due to that p53 is mutated in C33A (arg273cys) and wild type in the others. Wild type p53 is degraded by MDM2 or CHIP-mediated UPP which are inhibited when p53 is mutated [45–47]. The levels of Rap1GAP in HPV-positive cells did not increase, but decreased, after E6AP was knocked down, indicating that Rap1GAP could be degraded by other ways besides E6AP-mediated UPP. Autophagy is another important pathway of protein degradation, and there are complex cross-talks between autophagy and ubiquitination degradation pathways [48, 49]. We further explored whether knocking down E6AP activates autophagy to degrade Rap1GAP. The results showed that knocking down E6AP decreased the levels of p62 and increased the levels of LC3 II/LC3 I in HeLa cells but not in C33A cells (Fig. 5), suggesting that silencing of E6AP does activate autophagy in Hela cells but not in C33A cells. The results implicate that HR HPV infection plays a role in the selection of degradation pathways of Rap1GAP in which E6AP is involved.

Putting the above results together, we propose that Rap1GAP is degraded by the autophagy pathway in HPV-negative cells. When the cells are infected with HR HPVs, the viruses inhibit host autophagy to protect themselves from rapid degradation [50]. The HR HPVs employ E6AP to bind Rap1GAP, enabling Rap1GAP to be degraded by UPP. That is, the degradation of Rap1GAP is switched from the autophagy pathway to the E6AP-mediated UPP under HPV-infection-stress. Therefore, silencing of E6AP in HPV-positive cells will block the UPP degradation of Rap1GAP and restore the autophagy pathway, resulting in a decrease of Rap1GAP (Fig. 4A, B). Inducing autophagy will counteract the inhibition of UPP on the autophagy degradation of Rap1GAP in HPV-positive cells, leading to Rap1GAP degradation by autophagy (Fig. 6A). In HPV-negative C33A cells, because Rap1GAP is degraded by autophagy rather than UPP, silencing E6AP will not affect the degradation of Rap1GAP (Fig. 4C), while induction of autophagy leads to the degradation of Rap1GAP (Fig. 6B). The results also indicated that both HR HPVs infection and E6AP are indispensable for the switch. Rap1GAP is degraded by the autophagy pathway in the absence of HR HPVs, and switched to UPP degradation in the presence of HR HPVs. However, without E6AP, the UPP pathway for Rap1GAP degradation will be switched back to the autophagy pathway even in the presence of HR HPVs.

Rap1GAP is an important regulator of small G protein Rap1 activation. Our findings provide a better understanding of the mechanism of HPV-associated tumorigenesis and new potential targets for tumor therapy. The molecular mechanism through which HPV affects Rap1GAP degradation and its roles in cervical cancer development deserves further analysis.

## Conclusions

Rap1GAP may be degraded by autophagy, but HPV infection can turn Rap1GAP degradation from autophagy pathway to E6AP-mediated UPP, and E6AP may be one of the key components of the switch.

## Data Availability

The datasets used and/or analyzed during the current study are available from the corresponding author on reasonable request.

## Abbreviations

Co-IP: Co-immunoprecipitation
DMEM: Dulbecco’s modified Eagle’s medium
E1: Ubiquitin-activating enzyme
E2: Ubiquitin-conjugating enzyme
E3: The ubiquitin ligase
E6AP: E6-associated protein
ECL: Electrochemiluminescence
GAPDH: Glyceraldehyde-3-phosphate dehydrogenase
HECT: Homologous to E6AP C terminus
HR HPV: High-risk human papillomavirus
IP: Immunoprecipitation
MDM2: Murine double minute2
MEM: Minimum essential medium
MG132: Carbobenzoxy-leucyl-leucyl-leucinal
PVDF: Polyvinylidene fluoride
Rap1GAP: Ras-related regulatory protein 1 GTPase-activating protein
RING: Really interesting new gene
SDS-PAGE: Sodium dodecyl sulfate polyacrylamide gel electrophoresis
Ub: Ubiquitin
Ub(n): Polyubiquitin
UPP: Ubiquitin-proteasome pathway
UPS: Ubiquitin-proteasome system
WB: Western blotting
